# Machine-Learning Analysis of Serum Proteomics in Neuropathic Pain after Nerve Injury in Breast Cancer Surgery Points at Chemokine Signaling via SIRT2 Regulation

**DOI:** 10.3390/ijms23073488

**Published:** 2022-03-23

**Authors:** Jörn Lötsch, Laura Mustonen, Hanna Harno, Eija Kalso

**Affiliations:** 1 Institute of Clinical Pharmacology, Goethe-University, Theodor-Stern-Kai 7, 60590 Frankfurt am Main, Germany; 2 Fraunhofer Institute for Translational Medicine and Pharmacology ITMP, Theodor-Stern-Kai 7, 60596 Frankfurt am Main, Germany; 3 Pain Clinic, Department of Anaesthesiology, Intensive Care and Pain Medicine, Helsinki University Hospital and University of Helsinki, 00029 Helsinki, Finland; laura.mustonen@helsinki.fi (L.M.); hanna.harno@hus.fi (H.H.); eija.kalso@helsinki.fi (E.K.); 4 Clinical Neurosciences, Neurology, Helsinki University Hospital and University of Helsinki, 00029 Helsinki, Finland; 5 SleepWell Research Programme, University of Helsinki, 00014 Helsinki, Finland

**Keywords:** pain, neuropathic pain, postoperative pain, patients, human research, proteomics, machine-learning, data science

## Abstract

Background: Persistent postsurgical neuropathic pain (PPSNP) can occur after intraoperative damage to somatosensory nerves, with a prevalence of 29–57% in breast cancer surgery. Proteomics is an active research field in neuropathic pain and the first results support its utility for establishing diagnoses or finding therapy strategies. Methods: 57 women (30 non-PPSNP/27 PPSNP) who had experienced a surgeon-verified intercostobrachial nerve injury during breast cancer surgery, were examined for patterns in 74 serum proteomic markers that allowed discrimination between subgroups with or without PPSNP. Serum samples were obtained both before and after surgery. Results: Unsupervised data analyses, including principal component analysis and self-organizing maps of artificial neurons, revealed patterns that supported a data structure consistent with pain-related subgroup (non-PPSPN vs. PPSNP) separation. Subsequent supervised machine learning-based analyses revealed 19 proteins (CD244, SIRT2, CCL28, CXCL9, CCL20, CCL3, IL.10RA, MCP.1, TRAIL, CCL25, IL10, uPA, CCL4, DNER, STAMPB, CCL23, CST5, CCL11, FGF.23) that were informative for subgroup separation. In cross-validated training and testing of six different machine-learned algorithms, subgroup assignment was significantly better than chance, whereas this was not possible when training the algorithms with randomly permuted data or with the protein markers not selected. In particular, sirtuin 2 emerged as a key protein, presenting both before and after breast cancer treatments in the PPSNP compared with the non-PPSNP subgroup. Conclusions: The identified proteins play important roles in immune processes such as cell migration, chemotaxis, and cytokine-signaling. They also have considerable overlap with currently known targets of approved or investigational drugs. Taken together, several lines of unsupervised and supervised analyses pointed to structures in serum proteomics data, obtained before and after breast cancer surgery, that relate to neuroinflammatory processes associated with the development of neuropathic pain after an intraoperative nerve lesion.

## 1. Introduction

Persistent postsurgical neuropathic pain (PPSNP), defined as pain caused by a lesion of the somatosensory system associated with the surgical procedure [1], poses clinical challenges due to its intensity, relative resistance to current pharmacologic treatments, and sensory changes in the associated surgical area. Its estimated prevalence in women operated for breast cancer is 29–57% [2]. The reasons why neuropathic pain develops in only some patients after a similar nerve lesion are being investigated in several lines of research [3]. “Omics” is an emerging field in (neuropathic) pain research [4], and factors relevant to neuropathic pain include genetics [5,6], epigenetics [7], immunologics [8], metabolomics [9], and proteomics [10].

Proteomics is an active research field in neuropathic pain [11] and the first results support its utility. A literature search of the PubMed database at https://pubmed.ncbi.nlm.nih.gov for “proteomics and (neuropathic pain) NOT review(PT)” on 27 January 2022, yielded 99 hits, with the earliest article being from 2003 [12]. Proteomics provides access to neuroinflammation that is important for healing and regeneration after surgery, but can also transition to maladaptive neuroinflammation and contribute to the development and maintenance of pain [13]. An imbalance of pro- and anti-inflammatory cytokines in blood, cerebrospinal fluid (CSF), or neural tissue can promote persistent pain by sensitizing nociceptive signaling [13,14,15]. Most studies so far have compared neuropathic pain patients with healthy controls. However, neuropathy as such can also associate with a different cytokine profile when compared with healthy controls. One previous study showed that blood cytokine profiles differed between patients having painful or painless peripheral neuropathies and healthy controls. Proinflammatory cytokines, such as interleukin IL-2 and tumor necrosis factor-alpha (TNF-α), were found to be two-fold higher in painful neuropathies than in both painless neuropathies and healthy controls. On the other hand, levels of anti-inflammatory cytokine IL-10 were two-fold higher in painless neuropathies than in both painful neuropathies and healthy controls. The levels of another anti-inflammatory cytokine, IL-4, were 20-fold higher in patients with painless and 17-fold higher in patients with painful neuropathy, compared with healthy controls, suggesting that neuropathy as such may associate with increased production of anti-inflammatory cytokines, probably as compensatory mechanism, which may be more effective in those who do not develop painful neuropathies [16,17,18]. Similarly, CSF concentrations of CXCL6, CXCL10, CCL8, CCL11, CCL23 and of LAPTGF-β1 were higher in patients with peripheral neuropathic pain after surgery or trauma than in controls [15].

Using this complex knowledge of protein markers of pain, the present study aimed to narrow the focus to those proteins that are regulated differently in patients in whom PPSNP develops compared with those who do not develop neuropathic pain, despite similar intraoperative nerve lesion. Although the above-mentioned proteins are inflammatory markers, and immunological processes appear to be a common feature of persistent pain across various conditions [19], the underlying pathologies may differ among the causes of neuropathic pain. Thus, it is particularly important to compare the changes between those who develop or do not develop painful neuropathies after similar insult, e.g., breast cancer surgery, in whom the cancer and its treatment may alter protein patterns in different ways and may suggest future targets for pharmacological therapy. Such a cohort was available in a recent study [3,20], from which blood samples had been secured for “omics” analysis before and 4–9 years after the surgery. For this purpose, the Proseek multiplex inflammation panel [21] was selected as a collection of inflammation- and immune-related proteins for which associations with pain or cancer had already been reported in other clinical settings [22,23]. The present analysis of these new proteomics data pursued the hypothesis that breast cancer surgery-related neuropathic pain is reflected in a specific proteomics pattern. The aim of the present analysis, based on data science and machine learning, was to identify proteins that are most informative in distinguishing patients with and without PPSNP after breast cancer surgery and, therefore, most relevant for the development of future therapeutic strategies.

## 2. Results

### 2.1. Participants and Descriptive Data

**Figure 1:**
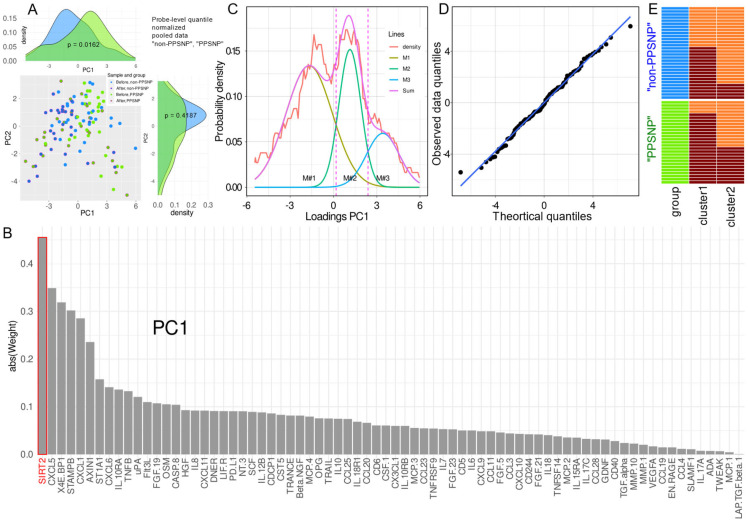
Results of a centered principal component analysis (PCA)-based projection of probe-level quantile normalized proteomics data normalization [32] acquired before (sample 1) and after the surgery (sample 2). (**A**): PCA-based projection of data set instances consisting of a sample in which d = 74 proteomic markers had been analyzed, with separations for acquisition time and patient subgroup (in blue color: no neuropathic pain, in green color: non-PPSNP, or neuropathic pain, PPSNP, despite intraoperative nerve injury). The marginal distribution plots show the segregation of predefined pain phenotype groups (non-PPSNP versus PPSNP) along the respective principal component. The *p*-values are the results from a Mann Whitney U-test [26,27], performed during “PC-corr” analysis [33] while attempting group segregation based on the respective PC. (**B**): Bar chart of the loadings of protein markers on PC1, sorted in descending order of magnitude. The proteins are named as in the Proseek panel for consistency. Please refer to Table 1 for standard protein names. (**C**): Distribution of the patients’ individual scores on PC1, described by the Pareto density estimation (PDE) [34], to which a Gaussian mixture model (GMM) with M = 3 modes was fitted. The Bayesian boundaries between the modes are indicated as dashed magenta perpendicular lines. The first boundary at x-position 0.18 provided a suitable GMM based grouping criterion of data set instances as shown in Panel E. (**D**): Quantile–quantile (QQ) plot of the theoretical and observed quantiles of the data, with line of identity. (**E**): Heatmap with the original subgroup structure (non-PPSNP versus “PPSNP”) and a subgroup structure that resulted from the GMM analysis of the coordinates of the projected samples on PC1 (see panel C). The color scheme green/blue of column 1 repeats that used in panel A for non-PPSNP versus “PPSNP “. The darker red color in columns 2 and 3 indicate data set instances belong to data set instances in Gaussian #1 of panel C, whereas the lighter orange color denotes data belonging to the second and third Gaussians combined in panel C. The GMM-based grouping significantly overlapped with the prior non-PPSNP versus PPSNP group structure (Fisher’s exact test [35]: *p* = 0.00468). The figure has been created using the R software package (version 4.0.2 for Linux; https://CRAN.R-project.org/ (accessed on 14 March 2022) [36]) and the library “ggplot2” (https://cran.r-project.org/package=ggplot2 (accessed on 14 March 2022) [37]).

In total, 251 patients with perioperative ICBN injury were assessed in the project [3]. According to generally accepted clinical criteria [24,25]. 31 patients fulfilled the criteria for PPSNP (NRS, 0–10) ≥4 whereas 34 patients had the nerve injury with no PPSNP or other chronic pain (non-PPSNP group, n = 34). Four patients were excluded from both groups: in the PPSNP group, three patients had metastasized cancer and one was an hs-CRP outlier; in the non-PPSNP group, two patients had metastasized cancer and two a chronic neurological disease. Thus, two groups of patients were analyzed comprising (i) “non-PPSNP” (n = 30), i.e., women who did not develop NP despite intraoperative nerve injury, and (ii) “PPSNP” (n = 27), i.e., women with NP after intraoperative nerve injury (Figure 1). 

The final PPSNP subgroup included 27 and the non-PPSNP group 30 patients. Before surgery, the patients in the non-PPSNP and PPSNP subgroups did not differ statistically significantly (Table 1) in age or body mass index (BMI); however, patients with PPSNP had slightly increased their BMI by the time when the second blood sample was collected. The interval between blood sampling was similar in non-PPSNP (7.8 ± 8.6 y) and PPSNP patients (5.2 ± 8.3 y; *t*-test: t = 1.1526, df = 54.721, *p*-value = 0.2541). A total of 8436 values of d = 74 different proteins were available from these patients. Descriptive statistics of the raw untransformed proteomics data and basic statistical assessments of group differences are given in Table 1.

**Table 1:** Baseline descriptive statistics of d = 74 proteomic markers recorded before or after surgery in patients who did not have persistent postsurgical pain (nonPPSNP, n = 30) or who had PPSNP (n = 27) 4–9 years after intraoperative nerve injury. Raw, i.e., untransformed data, and *p*-values of exploratory group-wise comparisons of proteomic markers are shown, separately for baseline or postoperative captures using Wilcoxon-Mann-Whitney U tests [26,27]. The proteins are named as in the Proseek panel. In addition, the standard names are provided along with the entry numbers in the National Center for Biotechnology Information (NCBI, Rockville Pike, Bethesda, MD, USA) [28] Entrez database at https://www.ncbi.nlm.nih.gov/Entrez/ (accessed on 14 March 2022), and the ID numbers in the Universal Protein Resource (UniProt) database at https://www.uniprot.org. (accessed on 14 March 2022) [29], queried using the R packages “annotate” (https://www.bioconductor.org/packages/annotate/ (accessed on 14 March 2022) [30]) and “org.Hs.eg.db”(https://bioconductor.org/packages/org.Hs.eg.db/ (accessed on 14 March 2022) [31]).

Protein				Baseline					Post-OP				
				Non-PPSNP		PPSNP			Non-PPSNP		PPSNP		
				Mean and SD	Range	Mean and SD	Range	Wilcoxon P	Mean and SD	Range	Mean and SD	Range	Wilcoxon P
**Demographics**													
**Age**				57.43 ± 7.84	33–68	53.85 ± 6.06	42–65	0.01941	64.03 ± 7.49	41–74	60.33 ± 5.84	48–71	0.01461
**BMI**				23.82 ± 3.52	17.8–30.8	25.22 ± 4.34	18.6–34.9	0.24	23.58 ± 3.72	16.8–30.12	25.97 ± 4.2	19.72–37.34	0.05718
**Proteins**													
**Variable name**	Standard names	NCBI	UNIPROT										
**ADA**	ADA	100	A0A0S2Z381	3.55 ± 0.41	3.09–5.1	3.64 ± 0.5	3.05–5.39	0.5307	3.71 ± 0.48	2.84–5.66	3.88 ± 0.65	3.14–6.03	0.5412
**AXIN1**	AXIN1	8312	A0A0S2Z4R0	2.86 ± 0.86	1.47–4.75	3.08 ± 0.57	1.62–4.05	0.1122	1.75 ± 0.99	0.55–4.29	1.84 ± 1.09	0.51–4.51	0.7936
**Beta.NGF**	NGF	841	A0A024R3Z8	1.5 ± 0.19	1.22–2.12	1.58 ± 0.39	1.3–3.06	0.8803	1.62 ± 0.27	1.23–2.22	1.6 ± 0.3	1.24–2.88	0.7331
**CASP.8**	CASP8	6356	P51671	0.93 ± 0.41	0.32–2.24	1 ± 0.58	0.34–2.55	0.8553	0.8 ± 0.55	0.16–2.99	0.98 ± 0.81	0.2–3.57	0.5951
**CCL11**	CCL11	6357	Q99616	7.49 ± 0.48	6.46–8.56	7.58 ± 0.44	6.9–8.41	0.5951	7.7 ± 0.52	6.66–8.73	7.85 ± 0.42	6.93–8.52	0.3051
**CCL19**	CCL19	6363	Q6IBD6	8.72 ± 0.85	7.3–11.21	8.91 ± 1.01	7.72–11.23	0.7936	8.54 ± 0.71	7.02–9.7	8.93 ± 1.06	7.67–12.26	0.2621
**CCL20**	CCL20	6364	P78556	4.71 ± 0.91	3.31–7.57	4.44 ± 0.92	3.2–6.76	0.1584	5.13 ± 0.89	3.83–7.97	4.84 ± 1.3	3.58–9.29	0.04785
**CCL23**	CCL23	6368	P55773	9.52 ± 0.33	9.05–10.46	9.34 ± 0.41	8.53–10.29	0.1017	9.39 ± 0.38	8.64–10.41	9.36 ± 0.44	8.52–10.24	0.6859
**CCL25**	CCL25	6370	O15444	6.02 ± 0.54	4.98–7.22	5.74 ± 0.51	4.49–6.67	0.0646	6.33 ± 0.61	5.01–7.79	6.02 ± 0.57	4.97–6.99	0.0773
**CCL28**	CCL28	56,477	A0N0Q3	1.49 ± 0.48	0.75–2.92	1.44 ± 0.34	0.78–2.34	0.9179	1.55 ± 0.52	0.91–3.63	1.4 ± 0.31	0.64–1.99	0.6512
**CCL3**	CCL3	6348	A0N0R1	4.23 ± 0.44	3.39–5.19	4.24 ± 0.44	3.39–4.87	0.9053	4.35 ± 0.47	3.58–5.26	4.52 ± 0.58	3.55–6.11	0.3051
**CCL4**	CCL4	6351	P13236	6.11 ± 0.46	5.33–7.21	6.24 ± 0.68	5.18–8.47	0.6512	6.15 ± 0.55	5.13–7.31	6.38 ± 0.69	5.29–8.45	0.2369
**CD244**	CD244	6354	P80098	5.49 ± 0.24	4.94–5.9	5.4 ± 0.28	4.72–5.99	0.2056	5.58 ± 0.3	5.05–6.08	5.58 ± 0.3	4.92–6.19	0.7451
**CD40**	CD40	6355	P80075	9.33 ± 0.31	8.69–10.44	9.3 ± 0.35	8.79–10.14	0.6061	9.37 ± 0.33	8.86–10.4	9.42 ± 0.4	8.76–10.66	0.6285
**CD5**	CD5	51,744	Q9BZW8	4.54 ± 0.3	4.05–5.25	4.53 ± 0.32	3.84–5.12	0.981	4.73 ± 0.32	4.2–5.46	4.74 ± 0.31	4.07–5.29	0.8429
**CD6**	CD6	29,126	Q0GN75	4.4 ± 0.31	3.75–5.1	4.5 ± 0.41	3.34–5.13	0.269	4.73 ± 0.45	3.83–5.64	4.8 ± 0.4	4.03–5.7	0.8305
**CDCP1**	CDCP1	958	A0A0S2Z3C7	2.99 ± 0.6	1.95–4.39	2.79 ± 0.4	1.96–3.54	0.2422	3.33 ± 0.62	2.33–4.61	3.31 ± 0.63	2.58–5.6	0.6398
**CSF.1**	CSF1	921	P06127	7.85 ± 0.17	7.56–8.18	7.82 ± 0.23	7.39–8.35	0.4899	7.93 ± 0.23	7.43–8.44	8.01 ± 0.2	7.66–8.41	0.2173
**CST5**	CST5	923	P30203	5.93 ± 0.43	5.27–7.23	5.87 ± 0.53	5.04–7.37	0.4225	6.18 ± 0.38	5.5–7.22	6.1 ± 0.56	5.11–7.34	0.3693
**CX3CL1**	CX3CL1	64,866	Q9H5V8	5.16 ± 0.3	4.46–5.66	5.04 ± 0.29	4.52–5.61	0.1446	5.26 ± 0.43	4.4–6.05	5.25 ± 0.35	4.6–5.83	0.9431
**CXCL1**	CXCL1	1435	A0A024R0A1	7.4 ± 1.04	5.24–9.44	7.43 ± 1.1	4.52–9.86	0.9684	6.71 ± 1.27	3.72–9.28	6.74 ± 1.57	3.75–9.05	0.7212
**CXCL10**	CXCL10	1473	P28325	7.43 ± 0.72	6.33–9.07	7.66 ± 0.88	6.56–9.92	0.3954	8.05 ± 0.6	6.92–9.57	8.27 ± 0.84	7.05–9.81	0.3779
**CXCL11**	CXCL11	1473	P28325	6.98 ± 0.94	5.57–8.73	7.1 ± 0.88	5.29–9.32	0.5732	7.22 ± 0.78	5.66–8.45	7.48 ± 1.09	5.69–9.82	0.3363
**CXCL5**	CXCL5	6376	A0N0N7	10.26 ± 1.41	7.24–13.22	10.32 ± 1.17	7.39–12.56	0.8678	9.49 ± 1.91	4.65–13.04	9.67 ± 1.61	5.9–12.5	0.6627
**CXCL6**	CXCL6	2919	P09341	6.98 ± 0.77	5.65–9.22	6.97 ± 0.75	5.37–8.5	0.9305	6.64 ± 0.77	4.93–8.86	6.8 ± 1.05	5.08–9.17	0.4701
**CXCL9**	CXCL9	3627	A0A024RDA4	7.2 ± 0.69	5.86–8.89	7.04 ± 0.63	5.8–8.65	0.3693	7.59 ± 0.7	6.57–8.89	7.52 ± 0.6	6.41–8.86	0.7571
**DNER**	DNER	6373	O14625	8.02 ± 0.22	7.46–8.44	7.98 ± 0.23	7.56–8.54	0.3779	8.1 ± 0.24	7.67–8.52	8.05 ± 0.23	7.58–8.46	0.4412
**EN.RAGE**	S100A12	6374	P42830	1.13 ± 0.51	0.24–2.83	1.22 ± 0.52	0.34–2.47	0.4899	1.24 ± 0.71	0.12–3.08	1.03 ± 0.57	0.3–2.74	0.1632
**FGF.19**	FGF19	6372	P80162	7.5 ± 0.96	5.92–10.58	7.53 ± 1	5.68–9.46	0.8059	7.73 ± 0.97	6.36–10.34	7.94 ± 0.81	5.91–9.43	0.1359
**FGF.21**	FGF21	3576	A0A024RDA5	5.93 ± 1.26	3.6–9.72	5.78 ± 0.93	3.87–7.72	0.6742	6.16 ± 1.12	3.64–8.14	5.87 ± 1.06	4.13–7.74	0.3363
**FGF.23**	FGF23	4283	Q07325	2.17 ± 0.48	1.51–3.63	2.06 ± 0.48	1.37–3.47	0.3363	2.38 ± 0.57	1.42–4.21	2.39 ± 0.44	1.77–3.48	0.8305
**FGF.5**	FGF5	92,737	Q8NFT8	1.13 ± 0.22	0.86–1.79	1.12 ± 0.19	0.67–1.47	0.8803	1.19 ± 0.21	0.87–1.81	1.16 ± 0.15	0.92–1.5	0.6976
**Flt3L**	FLT3LG	1978	Q13541	8.73 ± 0.3	8–9.29	8.71 ± 0.38	8.01–9.47	0.8553	9.25 ± 0.44	8.36–10.25	9.3 ± 0.48	8.41–10.67	0.6398
**GDNF**	GDNF	9965	O95750	1.23 ± 0.34	0.47–2.15	1.21 ± 0.3	0.64–2.24	0.7212	1.31 ± 0.36	0.64–2.14	1.26 ± 0.32	0.7–1.88	0.8305
**HGF**	HGF	26,291	Q9NSA1	8.89 ± 1.05	7.19–11.17	8.77 ± 1.02	7.26–10.52	0.7212	7.94 ± 0.46	7.2–9.01	7.98 ± 0.33	7.28–8.58	0.3779
**IL.10RA**	IL10RA	8074	Q9GZV9	1.35 ± 1.37	0.63–6.65	1.48 ± 0.8	0.63–3.99	0.03419	1.44 ± 1.39	0.63–6.51	1.47 ± 0.8	0.63–3.57	0.2843
**IL.10RB**	IL10RB	2250	Q8NBG6	6.43 ± 0.23	6.04–7	6.36 ± 0.26	5.87–6.91	0.3444	6.58 ± 0.29	5.77–7.31	6.62 ± 0.27	6.02–7.09	0.6061
**IL.12B**	IL12B	2323	B7ZLY4	4.08 ± 0.53	2.54–4.69	4.01 ± 0.63	2.78–5.14	0.4603	4.18 ± 0.51	3.12–5.28	4.11 ± 0.64	3.08–5.34	0.4799
**IL.15RA**	IL15RA	2668	A0A0S2Z3V2	0.03 ± 0.15	−0.23–0.31	−0.03 ± 0.17	−0.23–0.41	0.08132	0.1 ± 0.21	−0.23–0.56	0.1 ± 0.17	−0.23–0.59	0.8793
**IL.17A**	IL17A	3082	P14210	0.06 ± 0.54	−0.45–1.76	−0.01 ± 0.36	−0.45–0.98	0.6834	0.14 ± 0.6	−0.45–1.37	0.01 ± 0.48	−0.45–1.29	0.7789
**IL.17C**	IL17C	3586	P22301	0.65 ± 0.38	0.11–1.5	0.81 ± 0.57	0.11–2.6	0.3416	0.72 ± 0.41	0.11–1.54	0.65 ± 0.49	0.11–1.91	0.2878
**IL.18R1**	IL18R1	3587	Q13651	6.47 ± 0.32	5.77–7.04	6.44 ± 0.32	5.55–7.08	0.6512	6.55 ± 0.37	5.79–7.32	6.69 ± 0.41	6.01–7.74	0.3205
**IL10**	IL10	3588	Q08334	2.32 ± 0.44	1.35–3.54	2.16 ± 0.4	1.13–2.94	0.3205	2.53 ± 0.66	1.64–4.76	2.48 ± 0.5	1.75–4.08	1
**IL18**	IL18	3593	P29460	7.64 ± 1.01	6.64–12.33	7.37 ± 0.48	6.61–8.37	0.3526	7.53 ± 0.55	6.34–8.45	7.65 ± 0.47	6.78–8.62	0.4412
**IL6**	IL6	3601	Q13261	2.98 ± 1.14	1.54–6.45	2.67 ± 0.95	1.57–5.25	0.2295	3.09 ± 0.67	1.94–4.99	3.17 ± 1.04	1.72–6.94	0.7692
**IL7**	IL7	3605	Q16552	3.44 ± 0.76	2.44–5.49	3.2 ± 0.67	2.13–4.6	0.2903	2.9 ± 0.84	1.64–5.67	2.94 ± 0.64	1.79–4.02	0.6285
**IL8**	CXCL8	27,189	Q9P0M4	4.95 ± 0.63	4.09–6.43	4.9 ± 0.85	3.84–8.27	0.5732	4.83 ± 0.59	3.6–6.02	4.94 ± 0.71	3.62–6.22	0.6627
**LAP.TGF.beta.1**	TGFB1	3606	A0A024R3E0	6.82 ± 0.4	6.19–7.77	6.76 ± 0.35	6.06–7.65	0.6976	6.84 ± 0.43	5.79–7.99	6.9 ± 0.36	6.25–7.86	0.7094
**LIF.R**	LIFR	8809	Q13478	2.65 ± 0.23	2.15–3.04	2.62 ± 0.22	2.19–3.29	0.3363	2.74 ± 0.31	2.14–3.22	2.79 ± 0.2	2.47–3.19	0.7571
**MCP.1**	CST5	3569	B4DVM1	9.42 ± 0.35	8.76–10.04	9.52 ± 0.44	8.84–10.63	0.5101	9.63 ± 0.51	8.35–10.57	9.75 ± 0.39	9.06–10.63	0.3609
**MCP.2**	CCL8	3574	A8K673	7.53 ± 0.63	5.57–8.67	7.52 ± 0.57	6.12–8.41	0.8928	7.62 ± 0.7	5.74–9.13	7.59 ± 0.7	6.05–8.81	0.981
**MCP.3**	CCL7	4254	A0A024RBC0	0.87 ± 0.51	0.17–2.45	1.02 ± 1.11	0.06–6.02	0.9053	0.97 ± 0.52	0.1–2.06	1.16 ± 0.58	0.25–2.37	0.2234
**MCP.4**	CCL13	3977	A8K1Z4	3.46 ± 0.83	2.14–5.95	3.46 ± 0.48	2.69–4.61	0.7814	3.44 ± 0.73	2.2–5.01	3.56 ± 0.75	1.74–4.93	0.4318
**MMP.1**	MMP1	4049	P01374	12.2 ± 1.55	9.13–14.97	12.18 ± 1.27	9.96–13.93	0.9053	12.32 ± 1.22	10.56–14.34	12.27 ± 1.38	10.16–14.73	0.7936
**MMP.10**	MMP10	4312	B4DN15	5.62 ± 0.82	4.59–7.86	5.59 ± 0.75	4.57–7.55	0.8429	5.73 ± 0.7	4.91–7.71	5.8 ± 0.69	4.65–7.32	0.4701
**NT.3**	NTF3	4319	P09238	1.08 ± 0.64	0.46–3.35	0.97 ± 0.25	0.33–1.5	0.9179	1.2 ± 0.6	0.25–3.77	1.11 ± 0.55	0.64–3.58	0.1236
**OPG**	TNFRSF11B	4803	P01138	10.25 ± 0.4	9.39–11.01	10.12 ± 0.31	9.68–10.91	0.09834	10.37 ± 0.45	9.29–11.11	10.36 ± 0.3	9.93–11.22	0.6742
**OSM**	OSM	4908	P20783	2.48 ± 0.79	1–4.52	2.64 ± 0.95	0.37–4.14	0.3127	1.96 ± 0.66	0.82–3.24	2.22 ± 0.73	0.44–3.73	0.1359
**PD.L1**	CD274	5008	B5MCX1	3.39 ± 0.3	2.9–4.07	3.32 ± 0.27	2.79–3.81	0.9431	3.5 ± 0.52	2.65–5.51	3.48 ± 0.33	2.8–4.13	0.8429
**SCF**	KITLG	5328	P00749	9.49 ± 0.48	8.08–10.08	9.53 ± 0.44	8.14–9.98	0.9557	9.64 ± 0.31	8.94–10.25	9.68 ± 0.32	8.99–10.26	0.4507
**SIRT2**	SIRT2	6283	P80511	2.61 ± 1.18	1.11–6.12	3.12 ± 1.1	0.83–5.37	0.05166	1.89 ± 1.27	0.71–5.38	2.58 ± 1.9	0.54–8.22	0.1782
**SLAMF1**	SLAMF1	22,933	A0A0A0MRF5	1.2 ± 0.56	0.32–3.31	1.21 ± 0.89	0.49–5.24	0.3609	1.41 ± 0.67	0.2–3.97	1.48 ± 0.74	0.34–4.32	0.8305
**ST1A1**	SULT1A1	6504	Q13291	1.21 ± 0.82	−0.03–3.21	1.21 ± 0.59	0–2.1	0.6398	0.39 ± 0.64	−0.13–2.72	0.38 ± 0.69	−0.13–2.31	0.5136
**STAMPB**	STAMBP	10,617	A0A140VK54	4.22 ± 0.79	3.22–6.65	4.55 ± 0.73	2.93–6.17	0.1051	3.84 ± 0.98	2.83–7.12	4.2 ± 1.36	3–8.56	0.3693
**TGF.alpha**	TGFA	6817	P50225	2.72 ± 0.37	2.17–3.94	2.74 ± 0.4	1.88–3.66	0.7692	2.57 ± 0.46	1.82–4.45	2.47 ± 0.28	1.81–2.91	0.6742
**TNFB**	LTA	7039	P01135	3.65 ± 0.93	2.49–7.66	3.47 ± 0.41	2.64–4.27	0.7692	3.65 ± 0.44	2.59–4.65	3.68 ± 0.37	2.91–4.35	0.6859
**TNFRSF9**	TNFRSF9	7040	P01137	5.74 ± 0.37	5.26–6.74	5.62 ± 0.38	5–6.49	0.2114	5.96 ± 0.42	5.18–7.07	5.95 ± 0.38	5.22–6.56	0.9557
**TNFSF14**	TNFSF14	4982	O00300	3.9 ± 0.46	2.99–4.99	4.02 ± 0.49	2.93–4.93	0.2621	3.86 ± 0.51	2.94–4.77	3.99 ± 0.56	3.06–5.22	0.4225
**TRAIL**	TNFSF10	3604	Q07011	8.19 ± 0.54	7.39–10.41	7.95 ± 0.23	7.45–8.42	0.02957	8.35 ± 0.53	7.68–10.64	8.25 ± 0.18	7.95–8.61	0.8429
**TRANCE**	TNFSF11	8743	P50591	3.9 ± 0.62	2.77–5	3.97 ± 0.68	2.28–5.31	0.7212	4.19 ± 0.74	3.02–6.08	4.05 ± 0.53	3.1–5.46	0.4799
**TWEAK**	TNFSF12	8600	O14788	9.48 ± 0.49	8.62–10.38	9.41 ± 0.59	8.52–10.66	0.4999	9.1 ± 0.34	8.05–9.69	9.1 ± 0.3	8.4–9.71	0.9053
**uPA**	PLAU	8742	O43508	9.95 ± 0.33	8.97–10.46	9.79 ± 0.26	9.23–10.28	0.0168	10.26 ± 0.38	9.41–10.97	10.14 ± 0.31	9.43–10.62	0.276
**VEGFA**	VEGFA	8740	O43557	9.15 ± 0.47	8.27–10.01	9.01 ± 0.33	8.32–9.87	0.3205	9.24 ± 0.42	8.47–10.33	9.24 ± 0.41	8.5–10.32	0.8182
**X4E.BP1**	EIF4EBP1	7422	A0A087WUD8	7.66 ± 1.14	5.5–11.07	8.09 ± 1.4	6.41–10.94	0.3526	7.73 ± 1.32	6.15–11.62	8.05 ± 1.66	6.08–11.69	0.6285

### 2.2. Data Projection-Based Protein Marker Patterns Relevant to Pain-Related Subgroup Separation

The results of PC-Corr analyses indicated that the non-PPSNP versus PPSNP subgroups were best separated when the entire data set recorded before and after surgery was projected onto a lower dimensional plane after probe-level quantile normalization [32] and centering of the data. Significant segregation of the two neuropathic-pain related subgroups was already observed along the first dimension of the PCA projection of the whole data set as mentioned above (PC1; Wilcoxon-Man-Whitney U test *p*-value < 0.05, AUC-ROC = 0.63, AUC-PR = 0.58), which explained 19.6% of the total variance in the proteomics data (Figure 1A). The protein with the largest contribution to PC1 was SIRT2 (Figure 1B). The distribution of the coordinates of the projection of the observations on PC1 was best described by a trimodal Gaussian mixture (Figure 1C), which showed no significant difference between the fitted and observed distributions (Kolmogorov-Smirnov test: *p* = 0.895) and an almost linear placement of the quantiles in the QQ plot (Figure 1D). Using the first Bayesian decision limit at x-position 0.18, the resulting two groups of n = 60 and n = 54 samples significantly overlapped with the predefined subgroup structure of non-PPSNP versus PPSNP (Figure 1E; Fisher’s exact test: odds ratio 4.28, 95% confidence interval, CI: 1.384–7.367, *p* = 0.00468). Consideration of the second Bayesian decision boundary did not yield further significant results and was therefore abandoned.

**Figure 2:**
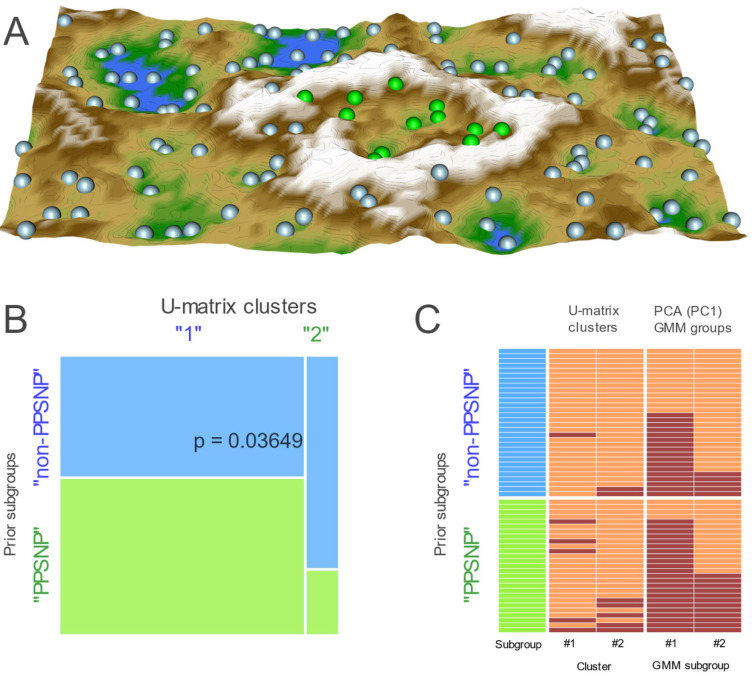
Results of projection of the data, after probe-level quantile normalization and pooled first and second samples, onto an emergent self-organizing map (ESOM; for further details of this artificial neuronal network-based data projection method, see [38,39]). (**A**): Three-dimensional U-matrix visualization of distance-based structures of the serum concentration of d = 74 proteomic markers following projection of the data points onto a toroid grid of 4000 artificial neurons where opposite edges are connected. The dots represent the so-called “best matching units” (BMU), i.e., neurons on the grid that, after ESOM learning, carried a data vector that was most similar to a subjects’ data vector. Please note that one BMU can carry vectors of several cases, i.e., the number of BMUs is not necessarily equal to the number of cases. The U-matrix visualization was colored as a top view of a topographic map with brown (up to snow-covered) heights and green valleys with blue lakes. Watersheds indicate borderlines between different clusters. Two clusters emerged in this way, separated by the white “mountain ridge” at the left of the U-matrix. BMUs belonging to clusters #1 or #2 are colored in green or bluish, respectively. (**B**): Mosaic plot, visualizing the contingency table between the original group structure and the cluster identified on the U-matrix. The *p* value of 0.03649 denotes the results of a Fisher’s exact test [35]. (**C**): Heatmap with the original subgroup structure (non-PPSNP versus “PPSNP”) and a subgroup structure that resulted from the U-matrix shown in in Panel A. The clusters based on the U-matrix (Panel A) are shown in the 2nd and 3rd column. For comparison, the PCA-based clusters (Figure 1D) are displayed in the last two columns The figure has been created using the R software package (version 4.0.2 for Linux; https://CRAN.R-project.org/ (accessed on 14 March 2022) [36]) and the libraries “ggplot2” (https://cran.r-project.org/package=ggplot2 (accessed on 14 March 2022) [37]), “ggmosaic” (https://cran.r-project.org/package=ggmosaic (accessed on 14 March 2022) [40]) and “Umatrix” (https://cran.r-project.org/package=Umatrix (accessed on 14 March 2022) [41]).

A subgroup structure as observed in the PCA-based projection of the proteomics data was further supported by an alternative projection on a trained emergent self-organizing feature map (ESOM). Large U-heights (Figure 2A) forming a “mountain ridge” separated a small region of 13 data points from the larger region of 101 samples, which indicated the emergence of two main clusters in the data. This agreed with the prior “non-PPSNP” and “PPSNP” group structure (Fisher’s exact test: odds ratio 4.26 (95% CI 1.017–25.55, *p* = 0.03649; Figure 2B). The separated subgroup was smaller than in the analogous PCA-based result; however, all contained data instances also in the smaller subgroups were separated from the majority on the PCA projection (Figure 2C).

### 2.3. Supervised Machine Learning-Based Identification and Evaluation of Proteomic Markers Informative for Pain-Related Subgroup Segregation

**Figure 3:**
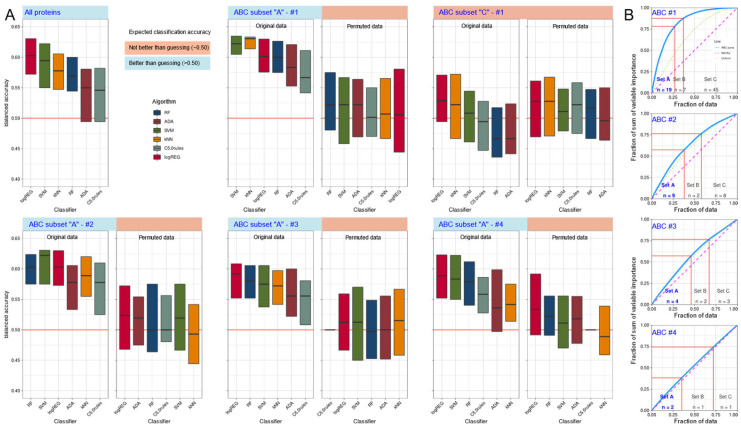
Results of supervised analyses of the possibility to train machine-learning algorithms with the information of selected proteomic markers to enable them to correctly assign a patient to the subgroup with nerve injury but without neuropathic pain (non-PPSNP) or to the subgroup with nerve injury and neuropathic pain (“PPSNP”). (**A**): Boxplots of the obtained balanced classification accuracy by different types of machine learning algorithms in assigning sub-jects to the subgroups when training was done with all protein markers or with the markers identified as the most informative in four consecutive item categorization techniques implemented as computed ABC analyses (for the protein markers identified as important, see Table 3). In case the selected proteins carried relevant information for patient subgroup assignment, the classification accuracy should be better than guessing. For comparison, the balanced classification accuracy achieved with permuted characteristics is shown, as well as the balanced classification (balanced) accuracy obtained when using the items placed by the first ABC analysis in subset “C”, which captures the least relevant items of a set. The expectations here were that without overfitting the classification (balanced) accuracy should not be better than guessing. The boxes have been constructed using the minimum, quartiles, median (solid line within the box), and maximum. The whiskers add 1.5 times the interquartile range (IQR) to the 75th percentile or subtract 1.5 times the IQR from the 25th percentile. (**B**): Results of the consecutive ABC analysis of the importance of protein markers. In the first ABC analysis, the counts were entered at which each maker occurred among the selected features in 1000 Boruta feature selection analyses on randomly drawn 2/3 of the data sets. In the subsequent ABC analyses, only the counts of occurrence of markers placed in ABC subset A by the previous ABC analysis were entered. The figure has been created using the R software package (version 4.0.2 for Linux; https://CRAN.R-project.org/ (accessed on 14 March 2022) [36]) and the R packages “ggplot2” (https://cran.r-project.org/package=ggplot2 (accessed on 14 March 2022)) and (https://cran.r-project.org/package=ABCanalysis (accessed on 14 March 2022) [42]).

**Figure 4:**
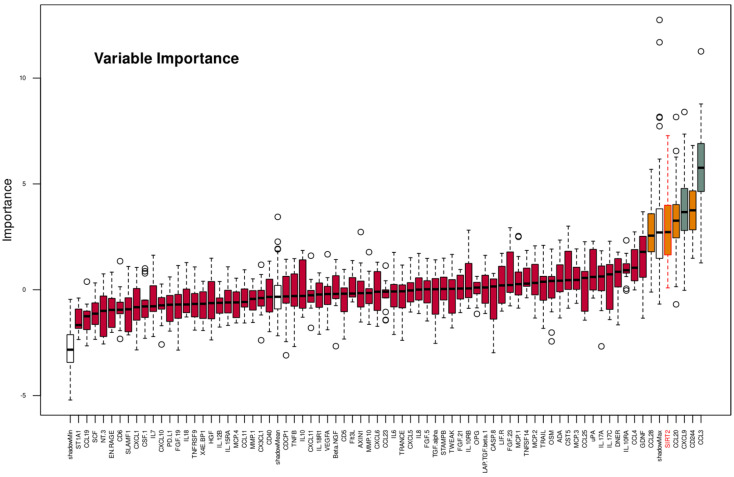
Example output of the importance analysis of protein markers for the allocation of patient subgroups (“non-PPSNP” versus “PPSNP”) according to an analysis based on random forests (“Boruta” [43]). The proteins are named as in the Proseek panel for consistency. Please refer to Table 1 for standard protein names. The importance measure of a feature (here: of the protein markers) results from the decrease in classification accuracy due to the random permutation of feature values. It is calculated separately for all trees in the forest that use the respective feature for classification. Then the mean value and the standard deviation of the loss of accuracy are calculated. The z-score is used in comparison to an external reference, the so-called “shadow” features, which is obtained by permuting the values of the original feature. The boxes were constructed using the minimum, quartiles, median (solid line inside the box) and maximum of these values. The whiskers add 1.5 times the interquartile range (IQR) to the 75th percentile or subtract 1.5 times the IQR from the 25th percentile. The black circles indicate outliers from this interval. The green and orange boxes represent “confirmed” or tentatively significant features, respectively, i.e., features that contribute to the classification success. The red boxes are confirmed as non-informative in order to be excluded from further analysis. The empty boxes are the above-mentioned “shadow” features. The figure has been created using the R software package (version 4.0.2 for Linux; https://CRAN.R-project.org/ (accessed on 14 March 2022) [36]) and the R library “Boruta” (https://cran.r-project.org/package=Boruta (accessed on 14 March 2022) [43]).

Training the classifiers with all d = 74 proteins included in this analysis was successful in logistic regression, support vector machine, k-nearest neighbors, and random forests, which were able to identify whether an instance of the dataset was acquired from a patient in the non-PPSNP or PPSNP subgroup (Figure 3A and Table 2). After feature selection using the Boruta method (Figure 4), d = 19 proteomic markers remained (Table 3). Training the classifiers with these d = 19 markers resulted in better classification performance than with all 74 markers, which is a typical observation in machine learning, where eliminating noise is often rewarded with better results. Now, all classifiers appeared to perform better than change in assigning a sample to the correct neuropathic pain subgroup. In contrast, when using permuted features or the d = 45 proteomic markers of ABC set “C,” i.e., the least important items, all classifiers resorted to random class assignment, indicating that (i) the successful classification results were unlikely to be due to overfitting and (ii) the item categorization captured the relevant items (Figure 3A).

Finally, to further narrow the focus on the most relevant proteomic markers, ABC analysis was performed in three further nested steps, whereby the feature set was successively reduced to d = 9, 4, and finally d = 2 protein markers (Table 3). This procedure can be repeated until the ABC curve (Figure 3B) touches the curve of uniform distribution of feature importance, since this curve marks the condition in which all features had the same chance to contribute to the subgroup separation, from which no particularly important feature can be separated any more. This procedure gradually reduced the classification power, but even with only CD244 and SIRT classification was still better than random assignment for logistic regression, support vector machine and random forests (Figure 3A). Of note, the observation of SIRT2 as the most prominent marker was consistent with its importance in the PCA projection on the most relevant PC1.

## 3. Discussion

The PPSNP and non-PPSNP subgroups showed different proteomics patterns when classical and machine learning-based feature selection techniques were used to identify the most informative proteins distinguishing these groups. The protein patterns already differed between the groups before nerve injury, whereas there was no clear difference when the proteins were compared before and after nerve injury. Thus, these distinct pre-injury protein patterns could reflect protective or predisposing factors associating with the development of PPSNP. The results of these analyses included 19 different serum protein makers from a candidate panel of 74 markers that could eventually be narrowed down to only two proteins with sitruin2 (SIRT2) as a possible predisposing protein for PPSNP. The present analyses were performed in the context of a concerted AI interpretation between data science and biomedical experts, as recently described [45], and conceptually similar to a conversational machine learning approach also recently presented [46], i.e., the results are facilitated by collaboration between different disciplines. Possible biomedical interpretations of the results are outlined below.

The NAD-dependent deacetylase sirtuin 2 (SIRT2) was identified as the most informative protein marker to train machine-learning algorithms to identify samples with neuropathic pain. SIRT2 is a class III histone deacetylase expressed ubiquitously, but more abundantly in the central nervous system than in other tissues [47]. It plays a role in microtubule acetylation and myelination [48], and it is involved in the suppression of NFkB-related inflammatory processes [49,50,51,52]. It is also involved in the regulation of neuroinflammatory processes via activation of microglia [53], which plays an important role in the response to peripheral nerve injury [54] and synaptic plasticity in persistent pain [55]. Another link to persistent pain arises from the role of SIRT2 in learning and memory, which are biological processes in terms of the Gene Ontology (GO) knowledgebase [56] and have emerged as key features of persistent pain in a computational functional genomics analysis [57]. SIRT2 is also involved in cancer where it has been proposed as both a tumor suppressor and tumor promoter [58]. However, its role as a tumor suppressor seems to be more frequently highlighted [59,60], and also in breast cancer [61]. It is also considered as a target for drugs against age-related and/or neurodegenerative disorders [62] and also for cancer [63].

A role of SIRT2 in neuropathic pain has been highlighted in a mouse model of cisplatin-induced peripheral neuropathy (CIPN) [64]. In humans, CSF-levels of SIRT2 were also among the protein markers relevant to persistent pain. Painful knee osteoarthritis has been patho-physiologically associated with neuroinflammatory processes and neuroimmune cross-links between the periphery and CNS. The CSF levels of SIRT2 were almost two-fold higher in the knee osteoarthritis patients than in healthy controls (See Table 3 in [65]). In the serum SIRT2 levels, however, there was no difference between the groups. In the present proteomics samples, the serum SIRT2 levels were higher in patients who developed neuropathic pain compared with those who had neuropathy without pain (Table 1). A brief review of what is known about SIRT2 in pain did not provide a clear direction of change. The cited results [65] in humans might be related to a pathology other than nerve lesion after surgery, whereas inflammation in arthritis and neuroinflammation in persistent pain represent a common mechanism. On the other hand, the rodent results are closer to the nerve lesion but were obtained in a laboratory model and in a different species, in contrast to the human origin and the real clinical setting in which both the arthritis study and the present study were performed.

SIRT2 is involved in the dynamics of the microtubule network in peripheral neurons, which forms the basis for axonal transport of proteins, RNA, vesicles, and organelles between the cell body and the axon tip [66]. It has been proposed that the dynamics of this network are maintained at an optimal level by the controlled action of tubulin-acetylating and -deacetylating enzymes [66]. SIRT2 belongs to the latter [67]. Lower tubulin acetylation is associated with lower microtubule stability [68] and lower recruitment of motor proteins to microtubules [69]. Therefore, high levels of SIRT2 in plasma could be a biomarker for lower microtubule acetylation associated with impaired axonal transport in peripheral neurons, and thus be causally involved in neuropathic pain. However, the enzymatic system that maintains the balance may overshoot, as has been shown in Charcot-Marie-Tooth neuropathy [66].

During the present analyses, SIRT2 was accompanied by a second marker, CD244, which remained among the selected features until the selection step (Table 3). CD244 is a cell surface receptor expressed on natural killer cells that activates cytotoxicity [70]. It has also been involved in cancer [71]; however, any direct involvement in pain has not yet been reported, although this is entirely conceivable via its immune modulation. In the present cohort, CD244 was higher in patients with neuropathic pain, which would be consistent with activated immune and inflammatory responses. The patients with painful knee arthrosis also had significantly higher CD244 levels compared with healthy controls in CSF, but not in serum (99).

**Figure 5:**
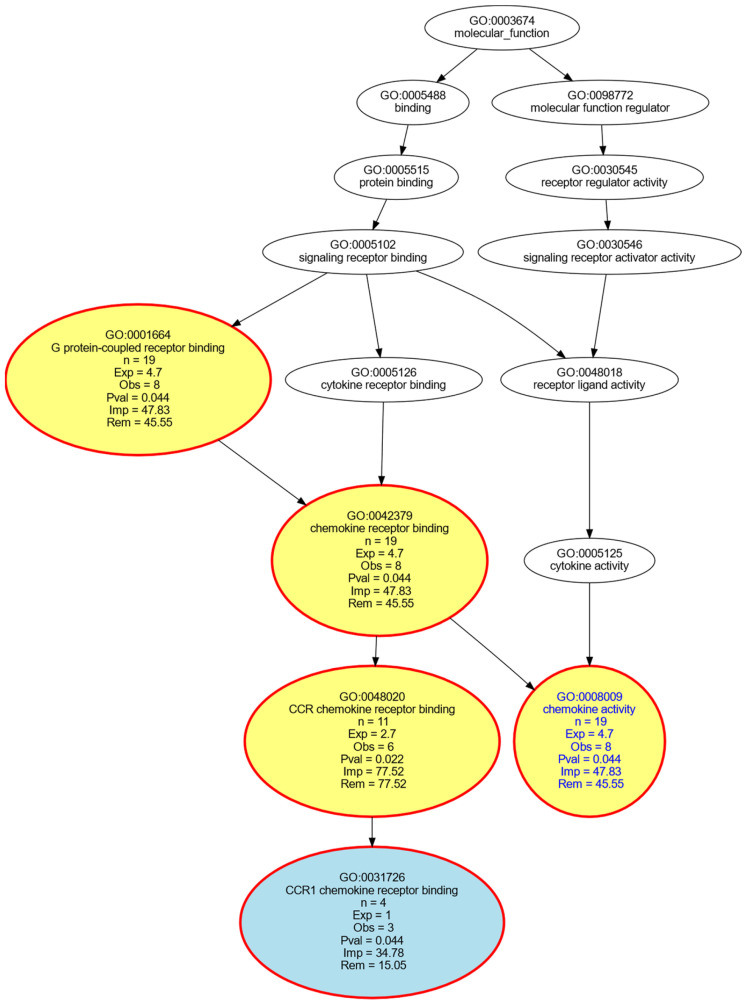
Computational functional genomics with respect to specific molecular functions in which the genes encoding the targets of the 19 proteins identified as informative for the presence or absence of neuropathic pain after nerve injury in breast cancer surgery are particularly involved among the genes encoding the entire Proseek multiplex inflammatory panel. The figure displays the results of an overrepresentation analysis (ORA; *p*-value threshold, tp = 0.05) of the n = 19 genes, contrasted with the genes encoding for the full Proseek panel, which served as reference gene set in the overrepresentation analysis. The graph shows the top-down representation of the annotations (GO terms) representing a systems biology perspective of the molecular functions modulated by the gene set. Each ellipse represents a GO term. The graphical representation follows the standard of the polyhierarchical organization of the GO knowledge base as a directed acyclic graph (DAG [62]). The color coding is as follows: No color: GO terms that are important for the DAG’s structure but do not have a significant *p*-value in Fisher’s exact tests. Red: Significantly overrepresented nodes. Green: Significantly underrepresented nodes. Blue: Terms at the end (detail) of a branch of the DAG. In addition, the node’s text will be colored in blue to indicate that this node is a detail. Yellow: Significant nodes with highest remarkableness in each path from a detail to the root, i.e., the so-called “headlines”. The figure has been created using the R software package (version 4.0.2 for Linux; https://CRAN.R-project.org/ (accessed on 14 March 2022) [36]) and the R library “dbtORA” (https://github.com/IME-TMP-FFM/dbtORA (accessed on 14 March 2022) [64]) with the DAG creation done with the GraphViz software package (https://graphviz.org (accessed on 14 March 2022) [77]).

Because the present analysis focused on reducing the Proseek multiplex inflammation panel [21] to the most relevant proteins associated with PPSNP after breast cancer surgery, it was important to define whether the selection represents, in functional terms, the entire panel or only proteins with specific molecular functions within the whole panel. To this end, an enrichment analysis was implemented as an overrepresentation analysis (ORA [72]) of the annotations to the genes encoding the selected proteins in the Gene Ontology (GO) knowledge base [56], where the current knowledge about genes is formulated using a controlled vocabulary of GO terms (categories) to which the genes [73] are annotated [74]. GO terms are related by “is-a”, “part-of”, and “regulates” relationships and form a poly-hierarchy represented as a directed acyclic (DAG [75]). The GO database can be searched by three main categories, namely biological processes, cellular components, and molecular functions. The GO category of molecular function, defined as molecular-level activities performed by gene products, such as “catalysis” or “transport” [56], was used as the functional selection of proteins was the main interest in this assessment. Hence, the 19 proteins identified as informative for the presence or absence of neuropathic pain after nerve injury in breast cancer surgery, were submitted to ORA with the whole Proseek multiplex inflammation panel as reference gene set. The analyses were carried out as described previously [76], using our R library “dbtORA” (https://github.com/IME-TMP-FFM/dbtORA (accessed on 14 March 2022) [77]), which in turn uses the data provided with the R packages “org.Hs.eg.db” (https://bioconductor.org/packages/release/data/annotation/html/org.Hs.eg.db.html (accessed on 14 March 2022) [31]) and “GO.db” (https://bioconductor.org/packages/release/data/annotation/html/GO.db.html (accessed on 14 March 2022) [78]) with the GO base version of 17 March 2021. For comparison, the full Proseek was analyzed against all human genes, using a *p*-value threshold of 0.05 and false discovery rate correction [79] for multiple testing performed by means of Fisher’s exact tests [35]. There, as a basis for selecting the most appropriate terms to describe the functional genomics roles of the genes of interest, so-called “headline terms” were used that to capture the main content of the poly-hierarchy resulting from ORA [80]. This analysis identified the terms GO:0098772 = molecular function regulator, GO:0005515 = protein binding, GO:0005488 = binding, GO:0060089 = molecular transducer activity, GO:0004175 = endopeptidase activity, GO:0008233 = peptidase activity and GO:0008236 = serine-type peptidase activity as the main molecular functions covered by the Proseek panel. Functionally contrasting the 19 genes coding for the 19 selected proteins with the genes coding the proteins of the whole panel was successful only when leaving out a correction; however, then a shift toward chemokines was observed with headline GO terms GO:0048020 = CCR chemokine receptor binding, GO:0001664 = G protein-coupled receptor binding, GO:0008009 = chemokine activity and GO:0042379 = chemokine receptor binding (Figure 5).

**Figure 6:**
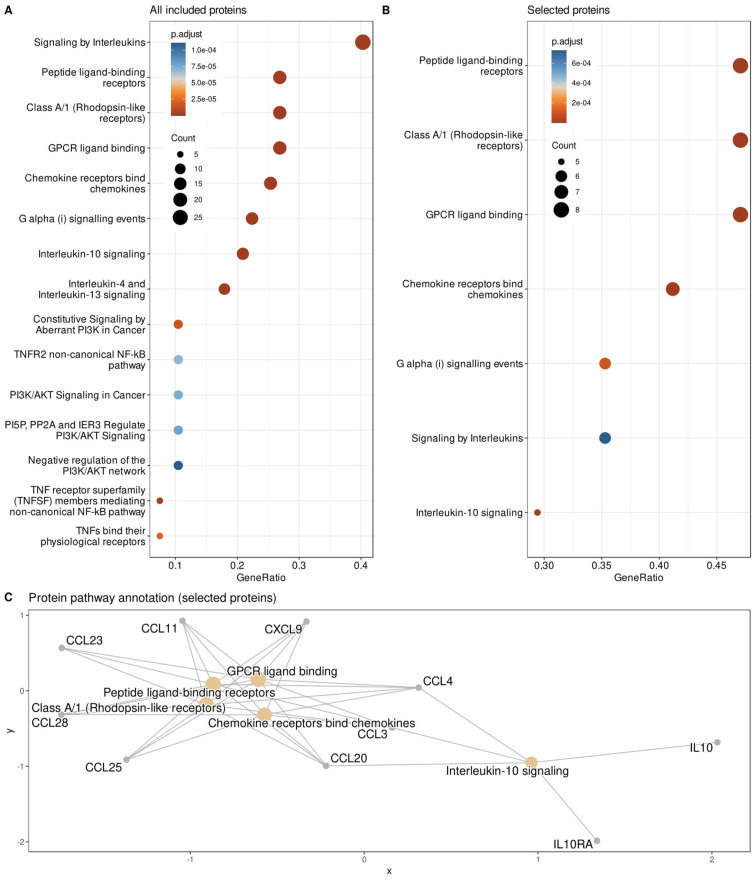
Reactome pathway-based analysis of the genes encoding the targets of the proteins identified as informative for the presence or absence of neuropathic pain after nerve injury in breast cancer surgery. (**A**): Pathways of the complete panel of proteins included in the present analyses. (**B**): Pathways of the genes encoding the targets of the 19 proteins identified as informative for the presence or absence of neuropathic pain after nerve injury in breast cancer surgery. (**C**): Network plot showing the biological complexities in which the genes belong to multiple annotation categories. The figure has been created using the R software package (version 4.0.2 for Linux; https://CRAN.R-project.org/ (accessed on 14 March 2022) [36]) and the R library “ReactomePA” (http://bioconductor.org/packages/release/bioc/html/ReactomePA.html (accessed on 14 March 2022) [81]).

In addition, the signaling pathways involving the currently analyzed proteins were assessed in a reactome pathway-based analysis using the R library “ReactomePA” (http://bioconductor.org/packages/release/bioc/html/ReactomePA.html (accessed on 14 March 2022) [81]) with its default parameter settings. This again pointed at chemokine signaling as also observed in the results of the above ORA, with the pathways involving the finally selected proteins including chemokine receptors bind chemokines, peptide ligand-binding receptors, interleukin-10 signaling, class A/1 (rhodopsin-like receptors), GPCR ligand binding, and G alpha (i) signaling events (Figure 6).

Further interpretation of the obtained results addressed the therapeutic potential of the present results, and known drugs were screened for an interaction with the d = 19 proteins of particular interest. This was done using the DrugBank database [82] at https://go.drugbank.com (version 5.1.8 dated 3 January 2021, accessed on 16 December 2021). The database was downloaded as an XML file (https://go.drugbank.com/releases/5-1-8/downloads/all-full-database, accessed on 14 March 2022) and processed using the R package “dbparser” (https://cran.r-project.org/package=dbparser (accessed on 14 March 2022) [83]). Cambinol is an experimental inhibitor of SIRT2 and is being investigated for use in cancer treatment. Any of the 19 proteins were listed as human targets for a total of 41 drugs, of which three were classified in the DrugBank as approved (amiloride, danazol, and chondroitin sulfate) and six were investigational drugs (fibrinolysin, ROX-888, CAT-213, CRx-139, LLL-3348, and again chondroitin sulfate), with the latter classified twice in the DrugBank. According to the DrugBank database, danazol is a steroid used to treat endometriosis and severe pain and tenderness associated with benign fibrocystic breasts, and chondroitin sulfate is used for osteoarthritis, which is also consistent with the overlap currently noted in the proteomics of both types of painful conditions. ROX-888 is being developed for severe acute pain and postoperative pain, CAT-213 is an antiallergic agent, and CRx-139 is being developed for the treatment of immune-inflammatory diseases, while LLL-3348 is intended for the treatment of psoriasis. Thus, the identified proteins point to very plausible drugs that clearly have a link to immunity, and the mention of pain among their possible clinical indications is also noteworthy.

The observed patterns in proteomics appeared to be present in both samples, i.e., those taken before surgery and chemotherapy and those at 4 to 9 years follow-up, although in the second sample the patterns associated with neuropathic pain appeared to be more pronounced. This could indicate protective or risk factors that the patients had already before surgery. It strengthens the association of the observed informative proteins with neuropathic pain and not with changes associated with time, different treatments, or cancer progression, which could have, though not specifically, accompanied the development of postoperative neuropathic pain between the two serum samples. However, the difficulties in observing clear differences between the preoperative and postoperative samples may also be related to the ultimately small sample size of the cohort. However, this is outweighed by the plausibility of the results, their partial replication of findings with persistent pain in independent cohorts, and their reflection in contemporary drug development activities. An independent verification of the present set of proteins most relevant to the development of neuropathic pain after intraoperative nerve injury in breast cancer will probably require a similar study, possibly with a narrower hypothesis that can be based on the present results, increasing the power of the study and possibly also enrolling a larger sample for this purpose. The present results are plausible in light of preclinical research, so return to preclinical models in rodents may not seem warranted. On the other hand, potential drugs resulting from the present findings may also need to be tested in patients, giving preference to experimental pain models in healthy subjects. That is, although systematic analyses have shown that experimental human pain models predict the clinical analgesic effects of drug candidates quite well when the right model for the clinical target is selected from a wide range of human experimental pain models [84,85,86], including models that appear to be predictive even for neuropathic pain drugs such as pregabalin [87], the complexity of the current clinical setting, including nerve injury and cancer treatment, may limit the utility of studies in healthy volunteers. However, depending on the particular characteristics and effects of a future new drug, it is difficult to predict the exact steps of drug development.

The present analyses were performed in serum, consistent with the increasing popularity of blood-derived biomarkers over CSF-derived markers as a more convenient and noninvasive approach for biomarker-based individualized prognosis and treatment of pain [88]. However, with the current analytical methods, the CSF samples are still more sensitive to detect differences in proteomics analyses when assessing pain associated with neuropathy (99). Since the present cohort consisted of women treated for breast cancer with drugs that promote peripheral nerve damage [89], the results need to be confirmed with larger cohorts of patients who do not have cancer.

## 4. Methods

### 4.1. Patients and Study Design

The Coordinating Ethics Committee of the Helsinki and Uusimaa Hospital District had approved the study, which was also registered at ClinicalTrials.gov (NCT02487524). All patients gave informed written consent. The study cohort consisted of a subset of patients from the NeuroPain study [3], which is a follow-up study of the original BrePainGen cohort in which perioperative pain and related psychological and genetic factors were examined in 1000 women undergoing surgery for breast cancer for unilateral, non-metastatic breast cancer at the Helsinki University Hospital between 2006 and 2010 [20]. Breast surgery consisted of either mastectomy or breast-conserving surgery with sentinel lymph node biopsy or axillary lymph node dissection. None of the patients had received neoadjuvant treatment. Postsurgical treatment consisted of chemotherapy, hormonal therapy and radiotherapy, according to the clinical guidelines.

Details of the clinical conditions and patient characteristics have already been described [3]. The NeuroPain cohort was recruited 4–9 years later in 2014–2016 from the BrePainGen cohort to study factors that associate with the development of neuropathic pain in patients who had a surgeon-verified complete or partial resection of the intercostobrachial nerve (ICBN) during surgery. The main inclusion criterion for the current sub-cohort was a surgeon-verified ICBN injury without persistent postsurgical neuropathic pain (non-PPSNP group) or with definite PPSNP and clinically meaningful pain intensity on a numerical rating scale (NRS, 0–10) ≥4, and no active cancer.

### 4.2. Acquisition of Pain-Related Information

At the preoperative visit, patients rated their pain during the past week in the area to be operated on and elsewhere, separately, on an 11-point numerical scale (NRS) (0 = no pain, 10 = worst pain imaginable). At the follow-up visit, sensory examination was performed to establish a diagnosis for PPSNP according to the latest NP grading criteria [1]. Other pain-related information collected from the patients included rating pain intensity on an 11-point numeric scale (0 = no pain, 10 = worst pain imaginable) by completing the Brief Pain Inventory (BPI) [90] for the worst pain experienced in the surgical area and elsewhere during the past week.

### 4.3. Blood Samples and Quantification of Serum Concentrations of Inflammatory Proteins

At the follow up visit, blood samples were collected for standard laboratory analysis of high-sensitivity C-reactive protein (hs-CRP) and oroso-mucoid (ORM), lipids (total cholesterol, high-density lipoproteins, low-density lipoproteins, and triglycerides), and 25-hydroxyvitamin-D). The results of these assessments have been reported previously [3].

For the proteomics analyses (Olink Analysis Service Uppsala, Uppsala, Sweden), blood samples were collected both before surgery in the BrePainGen study and at the follow up visit in ethylenediaminetetraacetic acid (EDTA) tubes and centrifuged at 3000 min^−1^ for 10 min. Serum was then transferred to cryotubes and the samples were immediately frozen and stored at −80 °C. The samples were collected and prepared by the same research nurse both preoperatively and at follow-up. The frozen samples were shipped on dry ice to Olink Proteomics, Uppsala, Sweden, for assay. The details of the assay have been described in detail by Wiberg et al. [91]. In brief, 92 proteins from the Proseek multiplex inflammation panel (https://bio-protocol.org/bio101/r9741259 (accessed on 14 March 2022) [21]) were quantified using a proximity extension assay (PEA) that involves two separate antibodies that bind to the same protein in a sample. Each antibody is coupled to a cDNA strand that is ligated on approach, extended by a polymerase, and finally detected using a Biomark HD 96 real-time dynamic PCR array (Fluidigm, South San Francisco, CA). Two incubation controls comprising green fluorescent protein and phycoerythrin were included in the assay to determine the lower limit of detection and to normalize the measurements. A normalized protein expression value (NPX) was calculated for each protein in the sample by normalizing the Ct values by subtracting the values for the extension control and an inter-plate control. The scale was shifted by a correction factor (normal background noise) [91]. Further details about initial laboratory data processing can be obtained at https://www.olink.com (accessed on 16 December 2021).

### 4.4. Data Analysis

#### 4.4.1. Summary of the Concept of Data Analysis

The goal of the study was to identify proteins from the Proseek multiplex inflammation panel that are most informative in discriminating patients with and without PPSNP after similar nerve injury during breast cancer surgery. The goal was translated into the task of “feature selection”, i.e., reducing data dimensionality by filtering out uninformative or redundant variables to simplify models for easier interpretation by field researchers [92]. Feature selection prior to training computational algorithms is a standard practice for improving classifier performance and reducing the computational burden of training and applying the algorithms. However, in addition to its main application of automatically assigning cases to classes or subgroups, supervised machine learning can also be used to discover structures in the data in order to obtain a description that provides better insights about the dataset. This knowledge discovery approach assumes that, if a classifier can be trained to identify whether a patient belongs to the PPSNP or non-PPSNP subgroup better than by guessing, then the features, i.e., the proteins in the dataset needed by the classifier to accomplish this task, contain relevant information about the addressed patient subgroup structure. In this way, the most informative proteins can be identified. In this use of feature selection, creating a powerful classifier is not the final goal, but feature selection takes precedence over classifier performance. This means that the analysis is considered as successful when the class assignment is just better than guessing and the variables needed for this assignment have been identified, and not necessarily that the classifier is further tuned.

Examples of feature selection methods [92] established in biomedical research [93] include classical approaches such as principal component analysis (PCA [94,95]), regression-based methods such as Least Absolute Shrinkage and Selection Operators (LASSO [96]), and methods based on generally well-performing machine learning methods such as the “Boruta” method [43] or an item categorization-based selection of the most important features for a classifier’s performance [97], both of which use the commonly used random forests machine learning classification algorithm as their basis [98,99]. For the present analysis, PCA and the “Boruta” method were used as a representation of a classical statistical approach and an established supervised machine learning approach. To evaluate whether the selected features actually contain information relevant to the subgroup structure in the present patient cohort, the identified features were then used to train a set of classifiers of different types, so as not to rely on the specifics of a single method, but to use a range of methods to internally validate the obtained results. The task here was to achieve better classification than random assignment to PPSNP or non-PPSNP subgroups, and this should not be similarly possible with the other proteins that were not selected as informative for this subgroup structure, nor should it be achieved when the classifiers were trained with permuted proteomics information, i.e., when the internal relationships of the protein levels to the pain-related subgroups were intentionally broken.

**Figure 7:**
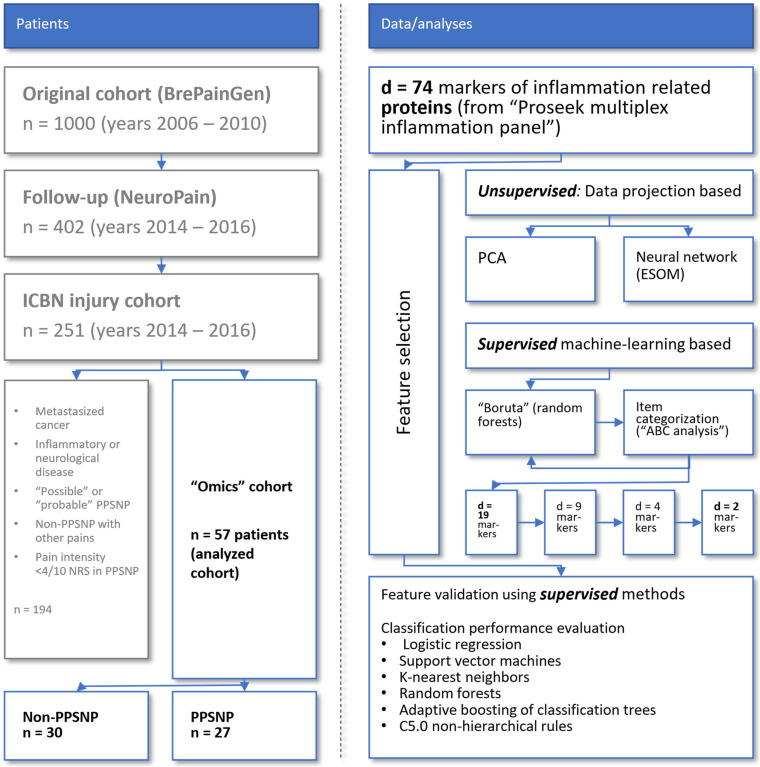
Flowchart showing the number of patients included in the different phases of the original study up to the present focused proteomics analysis. The figure has been created using Microsoft PowerPoint^®^ 365 (Redmond, WA, USA) on Microsoft Windows 11 running in a virtual machine powered by VirtualBox 6.1 for Linux (Oracle Corporation, Austin, TX, USA).

In its main components, the data analysis follows the previously proposed workflow for omics data from chronic patients [100] and is shown in a schematic drawing in Figure 7. The necessary programming work was performed in the R language [101] using the R software package [36], version 4.0.2 for Linux, which is available free of charge in the Comprehensive R Archive Network (CRAN) at https://CRAN.R-project.org/ (accessed on 14 March 2022). Analyses were performed on an Intel Core i7-10510U (Intel Corporation, Santa Clara, CA, USA) notebook computer running Ubuntu Linux 20.04.1 LTS 64-bit (Canonical, London, UK)). The detailed descriptions of the data analysis are provided in the following sections.

#### 4.4.2. Quantitative Information Analyzed

**Figure 8:**
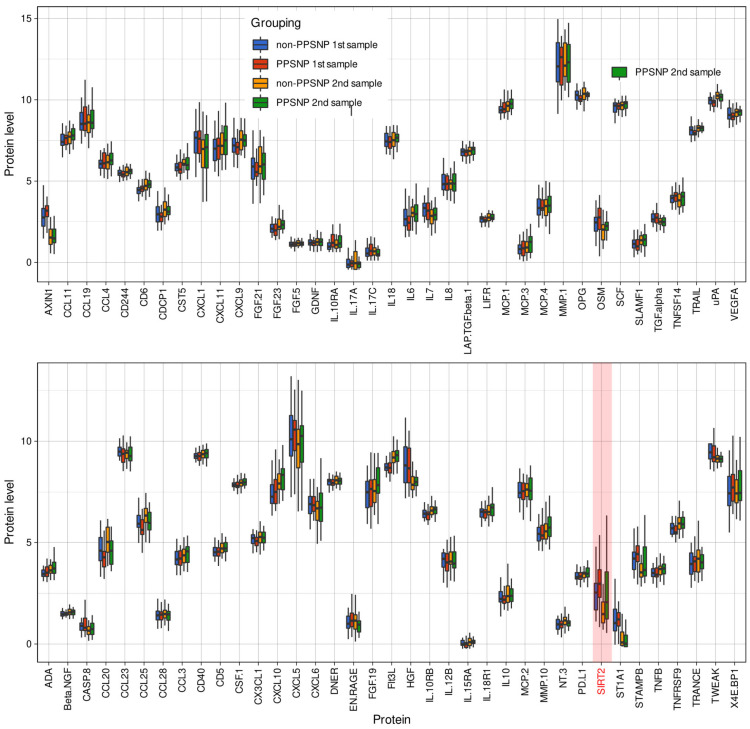
Plasma concentrations of protein markers. The proteins are named as in the Proseek panel for consistency. Please refer to Table 1 for standard protein names. The box plots show the raw values of proteomic marker levels in the plasma of the patients, separately for the first (before surgery) and second (at follow-up 4–9 years later) plasma sample and for the patients with nerve injury but no neuropathic pain (“non-PPSNP”) and patients with nerve injury in whom neuropathic pain developed “PPSNP”. The boxes were constructed using minimum, quartiles, median (solid line inside the box) and maximum. The whiskers add 1.5 times the interquartile range (IQR) to the 75th percentile or subtract 1.5 times the IQR from the 25th percentile. The presentation of the data has been arbitrarily split into two panels to enhance visibility. SIRT2 as a major result of the analysis is highlighted in red; for statistical details, see Table 1) The figure has been created using the R software package (version 4.0.2 for Linux; http://CRAN.R-project.org/ (accessed on 14 March 2022) [36]) and the R library “ggplot2” (https://cran.r-project.org/package=ggplot2 (accessed on 14 March 2022) [37]).

Pain-related information consisted of the presence or absence of PPSNP, scaled as [0, 1]. The proteomic panel included initially d = 92 different proteins [91]; however, d = 74 variables could be included in the analyses as the remaining proteins were below the detection level. The proteomic variables consisted of normalized serum protein expression value (NPX) [91], acquired before and 6.6 ± 1.2 (mean ± standard deviation) years after surgery. Thus the proteomic information provided a 74 × 114 (d × 2n) sized data space $D=\left\{\left(x_{i},y_{i}\right)|x_{i}\in \;{\mathbb{R}}^{X},\;y_{i}\in Y\left\{1,2\right\},\;i=1\ldots n\right\}$, which contained the information, x_i_ on d = 74 proteomic markers acquired at two time points from n = 57 patients, and an output data space, y_i_, that included the criteria for the grouping into two classes, i.e., the two patient groups comprising “nerve injury and no NP” (non-PPSNP)” and “nerve injury and NP” (PPSNP). The proteomics data set was complete and did not require imputations. Raw data, separated by subgroups and time of sampling, are shown in Figure 8.

#### 4.4.3. Data Projection-Based Assessment of Proteomics Data Structures Relevant to Pain-Related Subgroup Separation

PCA was performed using the recently proposed “PC-corr” approach [33]. This is an algorithm that facilitates PCA to find a data transformation that optimizes subgroup segregation by retrieving the correlations of the features that produce the segregation of the subgroups along a principal component (PC). It calculates different quality measures for each combination of PC, normalization and centering, and uses different transformations of the data. If its results consist of non-significant separations that are evaluated by quantitative analyses (expressed as *p*-value, AUC and AUPR) using any type of normalization and dimension, then a nonlinear dimensional reduction is required, since the data is difficult to linearize by different types of normalization. If it turns out that the significant separations, assessed by means of a Mann-Whitney U test [26,27], correspond to certain types of normalization and in dimensions that are not within the first three dimensions of the embedding, then the data has nonlinearities that can be treated by normalizing the data. Therefore, significant group separations in PC1-3 were sought in the PC-corr results as a basis for deciding on the most appropriate data transformation. This analysis was performed using an R script provided with the description of the PC-corr analysis (pccorrv2.R, https://github.com/biomedical-cybernetics/PC-corr_net (accessed on 14 March 2022) [33]). The results of this analysis indicated that the data set should be probe-level quantile normalized [32] for further analysis. This was performed using the R library “preprocessCore” (https://www.bioconductor.org/packages/release/bioc/html/preprocessCore.html (accessed on 14 March 2022) [102]).

In the relevant PCs resulting from the PCA described above, subgroup structures consistent with the prior classification (before versus after surgery, PPSNP versus non-PPSNP) were sought by means of Gaussian mixture modeling. Specifically, the distribution of the coordinates of the data set instances (observations) on the principal component space was described by the Pareto density estimation (PDE), which is a kernel estimator of the probability density function (PDF) that has been designed for group discovery [34]. Modal structures were analyzed by fitting Gaussian mixture models (GMM) to the PDE, using our interactive R tool “AdaptGauss” (https://cran.r-project.org/package=AdaptGauss (accessed on 14 March 2022) [103]). The quality of the fit was monitored using the root mean squares, and finally assessed using a Kolmogorov-Smirnov test [104] of the distribution of fitted versus observed data and visual inspection of the quantile-quantile plots of quantiles of the observed data versus the theoretical quantiles according to the fitted model. The assignment of subjects to the identified subgroups was determined using the Bayesian Theorem [105], which provides the decision limits for assigning a single observation to mode *M_i_* based on the calculation of posterior probabilities. The correspondence of the group assignment based on the Gaussian modes in the relevant PCs with the a priori subgroup distribution was statistically evaluated using Fisher’s exact tests [35].

As an alternative data projection method, self-organizing maps of artificial neurons were used [106] in a modification where the network consisted of a two-dimensional toroid grid with 50 rows and 80 columns [107] that has been shown to be well suited to subgroup detection in biomedical data [38]). Each neuron holds, in addition to a position vector on the two-dimensional grid, a further vector carrying “weights” of the same dimensions as the input dimensions. The weights were initially drawn randomly from the sets of data variables and subsequently adapted to the data during the learning phase with 20 epochs. Following training of the neural network, an ESOM was obtained that represented the subjects on a two-dimensional toroid map as the localizations of their respective “best matching units” (BMU). On the top of the obtained grid of trained neurons, the distances between the data points were calculated using the so-called U-matrix [39,108]. Each value (height) in the U-Matrix represents the average high-dimensional distance of one prototype in relation to all immediately adjacent prototypes in terms of grid position. The corresponding visualization technique uses a topographic map including the coloring, which facilitates the recognition of distance- and density-based structures. Large “heights” in brown and white colors represent large distances between the data. These calculations were performed using the R package “Umatrix” (https://cran.r-project.org/package=Umatrix (accessed on 14 March 2022) [41]).

#### 4.4.4. Supervised Machine-Learning Based Assessment of Proteomics Data Structures Relevant to Pain-Related Subgroup Separation

As an established method of feature selection in machine learning that precedes training of various different types of classifiers in different research environments, the random forest-based Boruta approach [43] was used to identify the most informative protein makers for partitioning the patient cohort into PPSNP and non-PPSNP subgroups. “Boruta” provides a decision on whether a variable is important or not for the classification task, which is derived from a 100-fold cross-validation approach followed by statistical evaluation of the variables importance with *p*-values defaulting to 0.01 [43]. These calculations were performed with the R package “Boruta” (https://cran.r-project.org/package=Boruta (accessed on 14 March 2022) [43]) with the default hyperparameter settings.

To further enhance the validity of the feature selection, the Boruta approach was nested into a 1000 cross-validation scenario using each time 2/3 of the data set randomly drawn class-proportionally from the original data set by means of using Monte Carlo resampling [109] implemented in the R library “sampling” (https://cran.r-project.org/package=sampling (accessed on 14 March 2022) [42]). The features selected by the Boruta algorithm during each run were collected, and the final set of proteins was assembled in descending order of the frequency with which they were among the selected features in the 1000 cross-validation Boruta runs. The cutoff value for the selection was set using the computed ABC analysis [110]. This item categorization method divides each set of positive numbers into three non-overlapping subsets “A”, “B”, and “C” [111], of which category “A” contains the “important few” that have been retained in the present analyses. The exact computations of the set limits “A/B” and “B/C” have been described elsewhere [110]; the calculations were performed using our R package “ABCanalysis” (https://cran.r-project.org/package=ABCanalysis (accessed on 14 March 2022) [110]).

#### 4.4.5. Supervised Machine Learning-Based Evaluation of Identified Proteomic Markers to Distinguish Pain-Related Patient Subgroups

The final step of the data analysis consisted in an evaluation of the identified proteomic markers to provide, in a variety of classification algorithms, suitable information about the segregation of the patient cohort into PPSNP or non-PPSNP subgroups. Classifier training and testing was performed in a 100-fold cross-validation design using disjoint training (2/3 of the data) and test (1/3 of the data) data subsets obtained by means of Monte-Carlo random resampling. Classification performance was evaluated primarily on the basis of balanced accuracy [112]. Further performance criteria included the area under the receiver operator curve (AUC-ROC [113]), sensitivity, specificity, precision, recall, positive and negative predictive value [114,115] and the F1 measure [116,117]. These calculations were performed with the R libraries “caret” (https://cran.r-project.org/package=caret (accessed on 14 March 2022) [118]) and “pROC” (https://cran.r-project.org/package=pROC (accessed on 14 March 2022) [119]).

The classifiers were trained with the selected proteomic markers, as these were of most interest in this evaluation of the results obtained in the previous steps of data analysis. If these markers enabled the algorithms to assign patients to pain subgroups better than by guessing, the selected proteins could be considered informative for this clinical subgrouping. To control for possible overfitting, all machine learning algorithms were additionally trained with randomly permuted proteomic markers, with the expectation that a classifier trained with these data should not perform better than guessing, i.e., give a balanced accuracy or an AUC-ROC around 50 %. Furthermore, classifiers were trained with all protein markers, and again with the protein markers that were not selected during feature selection, in order to ensure that the selection had indeed identified the most informative markers.

Supervised classification algorithms were chosen in order to cover a variety of machine learning classifiers, including (i) random forests [98,99], (ii) support vector machines (SVM [120]), (iii) adaptive boosting [121], (iv) k-nearest neighbors (kNN [122,123]), (v) C5.0 non-hierarchical rule-based classifier [124], and (vi) classical logistic regression [125]. The latter was preferred to classical alternatives consisting for example of linear discriminant analysis [126], following published advice on the choice between the two methods [127]. Moreover, in a direct comparison both methods have been shown to provide basically similar results on biomedical data [128]. For a review of machine learning methods that have been successfully applied to pain-related data, see e.g., [129]). The classifiers were available in the R libraries, “randomForest” (https://cran.r-project.org/package=randomForest (accessed on 14 March 2022) [130]), “xgboost” (https://cran.r-project.org/package=xgboost (accessed on 14 March 2022) [131]), “e1071” (https://cran.r-project.org/package=e1071 (accessed on 14 March 2022) [132]), “caret”, “C50” (https://CRAN.R-project.org/package=C50 (accessed on 14 March 2022) [133], and “nnet” (https://cran.r-project.org/package=nnet (accessed on 14 March 2022) [134]). Hyperparameters were tuned during grid searches. For example, random forests were built with 500 trees and *sqrt(d)* features per tree, SVM was executed with a linear kernel, while the k-nearest neighbors were used with centered and scaled prepossessed data, the Euclidean distance and the number of k = 3 for 10 or less features and k = 5 for >10 features.

## 5. Conclusions

Present analyses pointed in particular to sirtuin 2, with its role in neuroinflammatory processes and in learning and memory, as a key marker in the development of PPSNP. Results extended to 18 other proteins that were informative in distinguishing between samples from patients with neuropathic pain and those without neuropathic pain, without a clear distinction between samples before or after surgery. This suggests that the proteomic patterns were not simply a consequence of the development of neuropathic pain or other influences after surgery but reflected risk or protective factors that were already present before surgery. The identified informative proteins had a remarkable number of target proteins for approved or investigational drugs that have pain, including postoperative pain or chest pain, as a clinical target, providing remarkable support for the relevance of the present results.

## Figures and Tables

**Table 2:** Performance measures for the correct assignment of patients to the subgroup with nerve injury but without neuropathic pain (non-PPSNP) or to the subgroup with nerve injury and neuropathic pain (“PPSNP”). The performance of machine learning-based random forests classifiers is given; for further algorithms the key data (balanced accuracies) are shown in Figure 3. Classification performance was calculated (i) when training the algorithm with all protein markers or (ii–v) with the markers identified as the most informative in four consecutive item categorization techniques implemented as computed ABC analyses (“reduced data set #2–4; for the protein markers identified as important, see Table 3). For comparison, (vi) the balanced classification accuracy achieved with (permuted characteristics is shown, as well as the balanced classification accuracy obtained when using the items placed by the first ABC analysis in subset “C”, which captures the least relevant items of a set. For the protein markers identified as important, see Table 3). For comparison, the balanced classification accuracy achieved with permuted characteristics is shown, as well as the balanced classification accuracy obtained when using the items placed by the first ABC analysis in subset “C”, which captures the least relevant items of a set.

Parameter	Full Feature Set		Un-Selected Features		Reduced Set #1		Reduced Set #2		Reduced Set #3		Reduced Set #4	
**Protein #**	74		45		19		9		4		2	
**Data**	Original	Permuted	Original	Permuted	Original	Permuted	Original	Permuted	Original	Permuted	Original	Permuted
**Sensitivity**	65 (60–75)	65 (58.75–75)	57.5 (50–65)	60 (53.75–70)	70 (65–80)	60 (50–65)	70 (65–75)	55 (50–65)	65 (60–75)	65 (58.75–75)	70 (60–75)	55 (50–65)
**Specificity**	50 (44.44–61.11)	38.89 (27.78–44.44)	33.33 (27.78–38.89)	38.89 (27.78–44.44)	66.67 (55.56–72.22)	44.44 (33.33–50)	66.67 (55.56–72.22)	44.44 (33.33–55.56)	50 (44.44–61.11)	38.89 (27.78–44.44)	47.22 (38.89–61.11)	44.44 (33.33–55.56)
**Pos Pred Value**	60 (56.52–64.78)	54.01 (48.28–58.33)	48.15 (45.83–52.29)	52.51 (49.57–56.52)	68.83 (63.52–73.91)	53.39 (46.07–59.09)	68.42 (63.64–72.22)	52.63 (47.96–59.32)	60 (56.52–64.78)	54.01 (48.28–58.33)	60 (56–64)	53.85 (48.11–57.89)
**Neg Pred Value**	58.11 (52.86–65.42)	50 (40.88–58.33)	40 (35.71–46.84)	47.21 (41.18–53.33)	66.67 (62.35–71.63)	48.81 (38.37–55.73)	64.85 (61.05–70.15)	47.37 (40.88–55)	58.11 (52.86–65.42)	50 (40.88–58.33)	58.11 (53.24–63.8)	49 (40.91–53.33)
**Precision**	60 (56.52–64.78)	54.01 (48.28–58.33)	48.15 (45.83–52.29)	52.51 (49.57–56.52)	68.83 (63.52–73.91)	53.39 (46.07–59.09)	68.42 (63.64–72.22)	52.63 (47.96–59.32)	60 (56.52–64.78)	54.01 (48.28–58.33)	60 (56–64)	53.85 (48.11–57.89)
**Recall**	65 (60–75)	65 (58.75–75)	57.5 (50–65)	60 (53.75–70)	70 (65–80)	60 (50–65)	70 (65–75)	55 (50–65)	65 (60–75)	65 (58.75–75)	70 (60–75)	55 (50–65)
**F1**	63.29 (59.09–68.66)	59.09 (54.04–65.22)	53.2 (48.86–57.14)	56.52 (51.16–60.57)	69.77 (65.12–74.32)	56.47 (47.77–61.3)	68.36 (65.09–72.73)	55.16 (50–61.22)	63.29 (59.09–68.66)	59.09 (54.04–65.22)	63.53 (57.87–68.11)	55.68 (49.72–60)
**Prevalence**	52.63 (52.63–52.63)	52.63 (52.63–52.63)	52.63 (52.63–52.63)	52.63 (52.63–52.63)	52.63 (52.63–52.63)	52.63 (52.63–52.63)	52.63 (52.63–52.63)	52.63 (52.63–52.63)	52.63 (52.63–52.63)	52.63 (52.63–52.63)	52.63 (52.63–52.63)	52.63 (52.63–52.63)
**Detection Rate**	34.21 (31.58–39.47)	34.21 (30.92–39.47)	30.26 (26.32–34.21)	31.58 (28.29–36.84)	36.84 (34.21–42.11)	31.58 (26.32–34.21)	36.84 (34.21–39.47)	28.95 (26.32–34.21)	34.21 (31.58–39.47)	34.21 (30.92–39.47)	36.84 (31.58–39.47)	28.95 (26.32–34.21)
**Detection Prevalence**	60.53 (52.63–65.79)	65.79 (57.89–71.71)	61.84 (57.24–65.79)	60.53 (55.26–68.42)	55.26 (50–60.53)	57.89 (52.63–63.16)	52.63 (47.37–60.53)	57.89 (50–63.16)	60.53 (52.63–65.79)	65.79 (57.89–71.71)	60.53 (52.63–65.79)	55.26 (50–63.82)
**Balanced Accuracy**	57.92 (54.72–64.24)	51.81 (44.44–57.01)	44.44 (41.39–49.58)	49.86 (45.56–54.72)	68.33 (62.5–71.67)	50.97 (43.33–57.5)	66.25 (61.94–70.56)	50 (44.38–57.57)	57.92 (54.72–64.24)	51.81 (44.44–57.01)	59.17 (54.44–62.57)	51.25 (44.1–55.35)
**ROC-AUC**	57.92 (54.72–64.24)	54.44 (49.17–59.44)	49.72 (44.17–56.04)	52.5 (46.94–57.29)	68.33 (62.5–71.67)	55.56 (51.94–62.57)	66.25 (61.94–70.56)	54.72 (49.17–60.35)	57.92 (54.72–64.24)	54.44 (49.17–59.44)	59.17 (54.44–62.57)	54.86 (50–58.06)

**Table 3:** Details of the d = 19 proteins selected in a first computed ABC analysis that evaluated the counts at which each protein was among the selected features in 1000 Boruta feature selection analyses (Figure 4) on randomly drawn 2/3 of the data sets, aimed to identify the most relevant proteomic markers for assigning a patient to the subgroup with nerve injury but no neuropathic pain (non-PPSNP) or to the subgroup with nerve injury and neuropathic pain (“PPSNP”). The frequency occurrence in the set of selected features in the Boruta analysis is given in descending order. The *p*-values of group differences, calculated in the raw untransformed data, are the result of Mann-Whitney U tests [26,27], whereas the effect sizes of the group differences, quantified as Cohen’s d [44]. P-values in bold letters indicate significant effects for better visibility. Positive values indicate that the protein marker was observed at higher concentrations in the patients with neuropathic pain “(PPSNP”). The four consecutive ABC analyses reduced the feature set from the initial d = 19 proteins (all table) to finally d = 2 proteins (top two proteomic markers). The proteins are named as in the Proseek panel for consistency. Please refer to Table 1 for standard protein names.

ABC Subsets “A”				Proteomic Marker	Name	Gene Symbol	Frequency of Selection	Group Difference *p*-Value	Group Difference Cohen’s d
**ABC subset A” #1**	ABC subset A” #2	ABC subset A” #3	ABC subset A” #4	CD244	CD244 molecule	CD244	477	0.124	0.288
				SIRT2	Sirtuin 2	SIRT2	424	**0.0119**	0.49
				CCL28	C-C motif chemokine ligand 28	CCL28	409	0.203	−0.399
				CXCL9	C-X-C motif chemokine ligand 9	CXCL9	389	**0.0229**	−0.383
				CCL20	C-C motif chemokine ligand 20	CCL20	339	**0.0115**	−0.312
				CCL3	C-C motif chemokine ligand 3	CCL3	297	0.194	0.323
				IL.10RA	Interleukin 10 receptor subunit alpha	IL10RA	243	0.0647	0.037
				MCP.1	C-C motif chemokine ligand 2	CCL2	241	**0.0371**	0.452
				TRAIL	TNF superfamily member 10	TNFSF10	241	**0.0131**	−0.532
				CCL25	C-C motif chemokine ligand 25	CCL25	237	**0.027**	−0.469
				IL10	Interleukin 10	IL10	200	0.0814	−0.39
				uPA	Plasminogen activator, urokinase	PLAU	181	**0.0474**	−0.42
				CCL4	C-C motif chemokine ligand 4	CCL4	176	**0.036**	0.439
				DNER	Delta/notch like EGF repeat containing	DNER	146	**0.0205**	−0.392
				STAMPB	STAM binding protein	STAMBP	137	0.0803	0.394
				CCL23	C-C motif chemokine ligand 23	CCL23	113	0.0929	−0.339
				CST5	Cystatin D	CST5	111	0.788	0.123
				CCL11	C-C motif chemokine ligand 11	CCL11	108	0.275	0.252
				FGF.23	Fibroblast growth factor 23	FGF23	108	0.0676	−0.304

## Data Availability

The original data containing the patients’ proteomics information cannot be shared due to data security restrictions.
